# Face and face pareidolia in patients with temporal lobe epilepsy indicates different neural processing: an event-related potential study

**DOI:** 10.1186/s42494-024-00175-2

**Published:** 2024-10-09

**Authors:** Gülsüm Akdeniz, Sadiye Gumusyayla, Gonul Vural, Orhan Deniz, Pınar Özışık

**Affiliations:** 1 Department of Biophysics, Department of Neuroscience, Faculty of Medicine, Ankara Yildirim Beyazit University, and Yenimahalle Training and Research Hospital, Ankara, 06018 Turkey; 2 Department of Neurology, Faculty of Medicine, Ankara Yildirim Beyazit University, Ankara, 06801 Turkey; 3 Department of Neurosurgery, Faculty of Medicine, Ankara Yildirim Beyaziat University, Ankara, 06800 Turkey

**Keywords:** Face pareidolia, Pareidolia, Temporal lobe epilepsy, N170, Face, Neural processing

## Abstract

**Background:**

Visual perception of face images or face pareidolia can be evaluated with event-related potentials (ERP) for healthy subjects and patients with neurological conditions. In this study, we aimed to analyse event-related potential components such as P100, N100, N170, and vertex-positive potential (VPP) in response to face pareidolia perception in temporal lobe epilepsy (TLE) patients.

**Methods:**

ERPs were recorded during the pareidolia test. Waveforms were analzyed and current source density (CSD) maps were generated.

**Results:**

CSD profiles were shown to be interpretable when face and face pareidolia conditions. N100, P100, and N170 components showed larger amplitudes and longer latency in epilepsy patients in response to face pareidolia stimuli compared to real face images. However, the N170 component latency did not differ significantly between epilepsy patients and healthy participants, while the larger amplitude and longer latency of N100 and P100 responses were evoked in healthy patients.

**Conclusions:**

Our results indicate a difference in the neural mechanisms of processing real face information and pareidolia face-like information in TLE patients.

## Background

Epilepsy is a neurological condition characterized by the occurrence of spontaneous seizures and varying degrees of cognitive impairments, depending on the involvement of specific cortical networks in the pathology. In the field of epilepsy research, event-related potentials (ERPs) can directly measure brain activity and provide valuable insights into the pathogenesis and treatment of epilepsy [1, 2]. Moreover, ERPs can be used to assess cognitive dysfunction of epileptic patients and explore the underlying pathology [3].

Pareidolia refers to the phenomenon in which individuals perceive familiar patterns, such as faces, in random or ambiguous stimuli features [4]. The tendency to perceive meaningful patterns, particularly faces, in ordinary objects or abstract patterns is a fundamental aspect of human visual perception. Face pareidolia is a specific form of pareidolia characterized by the human tendency to perceive faces in inanimate objects [5]. Although pareidolia can create a strong impression of the presence of a face, it remains unclear to what extent these pareidolia faces are processed by the brain as actual faces [6]. The duration of epilepsy can impact the processing of faces. In patients with left-sided hippocampal sclerosis (HS), a shorter duration of epilepsy is linked to the activation in the left anterior hippocampus, while a longer duration of epilepsy is linked to frontal activation during the encoding of words and faces [7]. Understanding the mechanisms of pareidolia face encoding can provide valuable insights into how the brain perceives and recognizes faces, and shed light on the cognitive and social consequences associated with this phenomenon.

The P100 is one of the earliest components of ERPs, is a positive deflection that is primarily observed at the occipitotemporal region, typically peaking around 100–130 ms after presentation of a stimulus. A study has shown that the early P100 component is sensitive to changes in facial structure caused by face inversion [8]. The N100 is a negative evoked potential which occurs approximately simultaneously with P100 after stimulus onset. In the processing of facial expressions, the early anterior N100 component exhibits a larger response when presented with fearful faces compared to happy or neutral faces. This suggests the presence of a "negativity bias" in the processing of facial expressions, where the brain demonstrates a higher sensitivity towards negative emotional stimuli, particularly fearful faces [9]. The N170 component, which is associated with face processing, is qualitatively different from the N100 component associated with object processing [10]. The N170 is a negative component identified at the lateral occipitotemporal region and typically occurs in the 160–190 ms range, peaking around 170 ms. The N170 waveform reflects perception of faces from other visual stimuli and is considered a measure of the structural processing of faces [11–14]. The N170 component origins from the fusiform face area, located in the fusiform gyrus and superior temporal sulcus [10, 15–19]. Another positive component, known as the vertex positive potential (VPP), is observed at the frontocentral area and has a similar latency as the N170. Jeffreys found a higher latency of the face-specific VPP in response to inverted faces [20]. Previous studies showed that the VPP and the N170 are responsive to structural processing of faces [12, 21, 22]. Eimer suggested that the VPP and the N170 originated from identical neuronal regions in or near the fusiform gyrus [21]. In contrast, other researchers have claimed two independent neural regions for VPP and N170 [11, 21, 23, 24]. The N170 and VPP waves are associated with earliest sensory processing that distinguishes faces from objects [14, 25]. These two ERP components show greater sensitivity to faces compared to various object categories [10, 14, 15, 23, 26]. While the N170 has been found to be enhanced and delayed in response to scrambled faces, no significant modulation of the VPP has been observed [27]. Additionally, a study has demonstrated that inverted faces evoke a greater N170 response, but do not have a remarkable effect on the level of VPP [22].

A previous study has reported decreased N170 amplitude in patients with temporal lobe epilepsy (TLE) compared with normal controls, and an inversion effect for face stimuli (larger N170 amplitude in response to inverted faces than to upright faces) in both groups [28]. Additionally, another study demonstrated that the fusiform face area shows a greater response to upright faces compared to inverted faces [28–31]. These findings highlight the importance of understanding face and illusory face processing in epilepsy patients. However, there is limited research on the mechanisms of face pareidolia in epilepsy. In this study, we set out to explore the neural mechanisms of pareidolia in TLE patients by examining the P100, N100, N170, and VPP components of ERP.

## Methods

### Participants

This study recruited 30 individuals diagnosed with epilepsy (17 females, 13 males; mean age 33.6 ± 7.3 years) and 28 healthy participants (16 females, 12 males; mean age 28.4 ± 6.3 years). The demographic and clinical characteristics of the participants are presented in Table 1. Participants in the control group had no history of brain injury or neurological conditions. All participants had normal or corrected-to-normal visual acuity and no ocular pathology. This study was approved by the Ankara Yıldırım Beyazıt University Ethics Committee (No. 26379996/03). Written informed consent and publication consent were obtained from all participants.

**Table 1:** Demographic and clinical characteristics of the study groups (value)

	**TLE**	**Healthy group**
*n*	30	28
Gender (male/female)	13/17	12/16
Age, mean ± SD (years)	33.6 ± 7.3	28.4 ± 6.3
Disease duration (years)	7.93	NA
Laterality (right/left)	19/11	NA
MMSE, mean ± SD	27.8 ± 2.6	29.1 ± 1.0
Number of ASMs	1.7 ± 1.1	NA
Hippocampal sclerosis/normal	5/25	NA

*TLE* Temporal lobe epilepsy, *MMSE* Mini-Mental State Examination, *ASM* Anti-seizure medications, *NA* Not applicable

### Stimuli

**Fig. 1:**
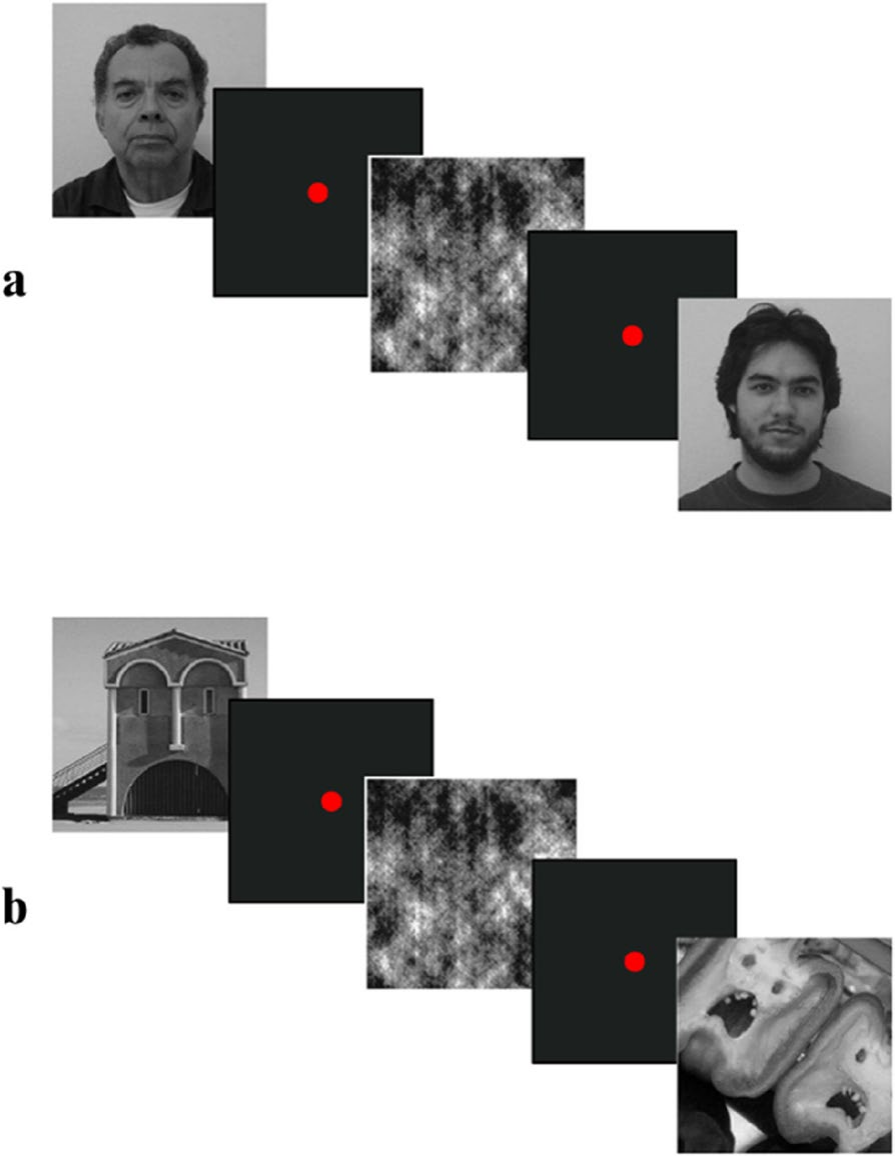
Examples of image presentation in the experiment. **a** In the real face perception test, a real face image is presented, followed by a small red circle that served as a fixation point and then a scrambled image. **b** In the pareidolia perception test, a face-like image is presented, followed by a small red circle that served as a fixation point and then a scrambled image

The face pareidolia images utilized in this study were sourced from the internet, while the face photos were obtained from the Centro Universitário da FEI [32]. The selected face photos were neutral in expression and devoid of any emotional states. In our previous study, we developed two versions of test, the face and the face pareidolia tests [33]. Scrambled images were created from face and face pareidolia photos using the SHINE toolbox of the Matlab software (MathWorks, Inc., Natick. MA) to serve as the non-targeted stimuli. The stimulation levels of face or face-like images were parametrically modified by the SHINE (spectrum, histogram, and intensity normalization and equalization) toolbox to equate them in the corresponding image type, thereby minimizing potential confounding effects of stimulation level. The experiment consisted of 60 visual stimuli, comprising 20 face images, 20 scrambled images, and 20 face pareidolia images (Fig. 1). All images were converted to grey scale with same resolution, position, luminance, and contrast. The visual stimuli were standardized to a dimension of 384 × 384 pixels.

### Procedure

During a single laboratory visit, participants were subjected to two experimental blocks as part of the stimulus sessions. A total of 60 stimuli were used, of which 20 were pareidolia images, 20 were face images, and 20 were scramble images. The same scramble images were used in both blocks. The presented images were displayed in a random order, with each image being shown for a duration of 1000 ms, followed by an interval of 1000 ms. The images were presented on a 19-inch monitor positioned at a distance of 1 meter from the participant. To avoid eye movement, a small red circle was presented on the monitor as a fixation point. The participant and the monitor were positioned at a 180-degree angle. Participants were asked to press a button when they perceived any real face or face-like images. To inform participants to understand the concept of pareidolia, a brief trial consisting of 5 pareidolia images was presented, providing them with visual information about the nature of pareidolia.

### EEG recording

**Fig. 2:**
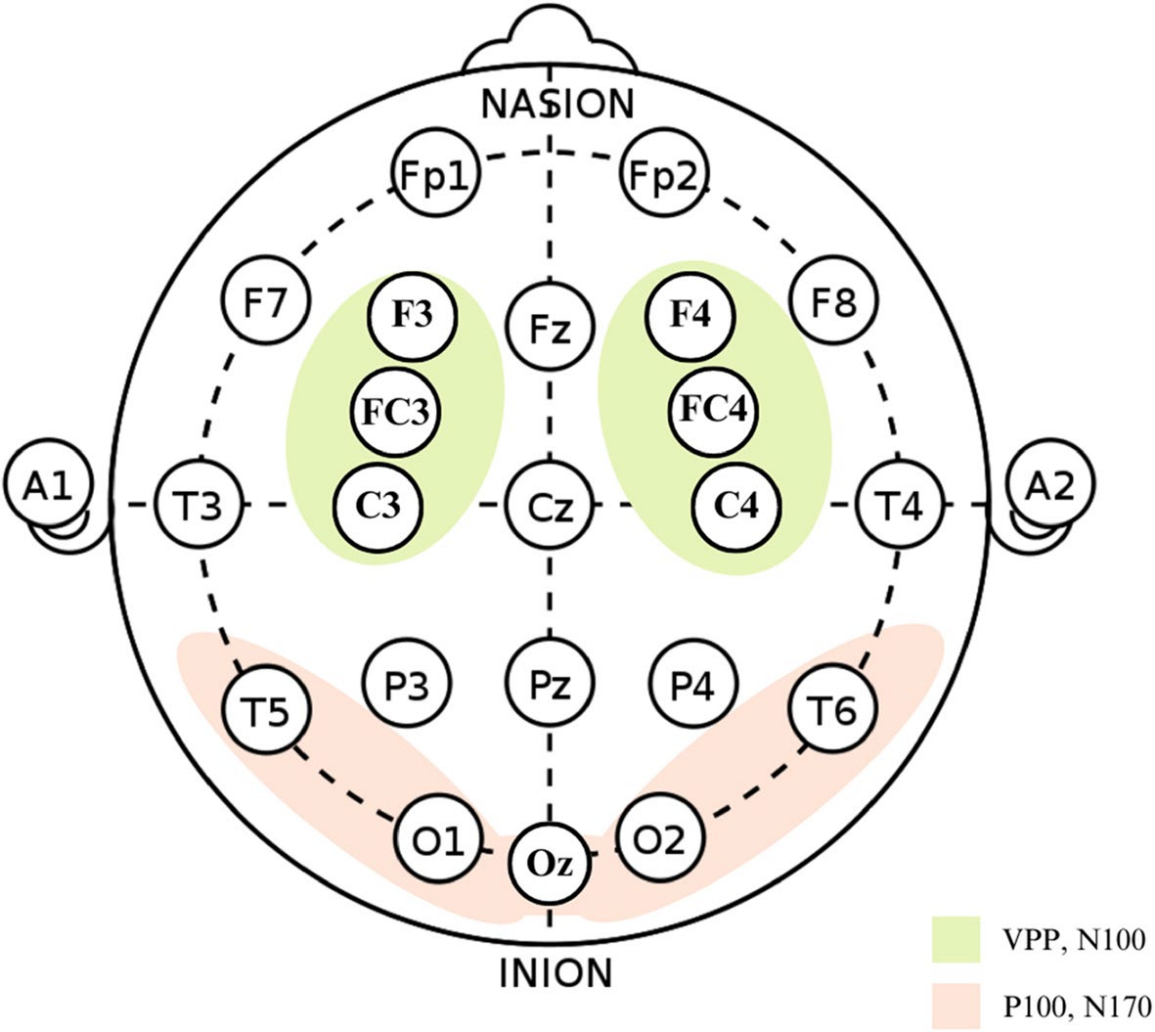
An illustration of electrode sites of ERP components

**Fig. 3:**
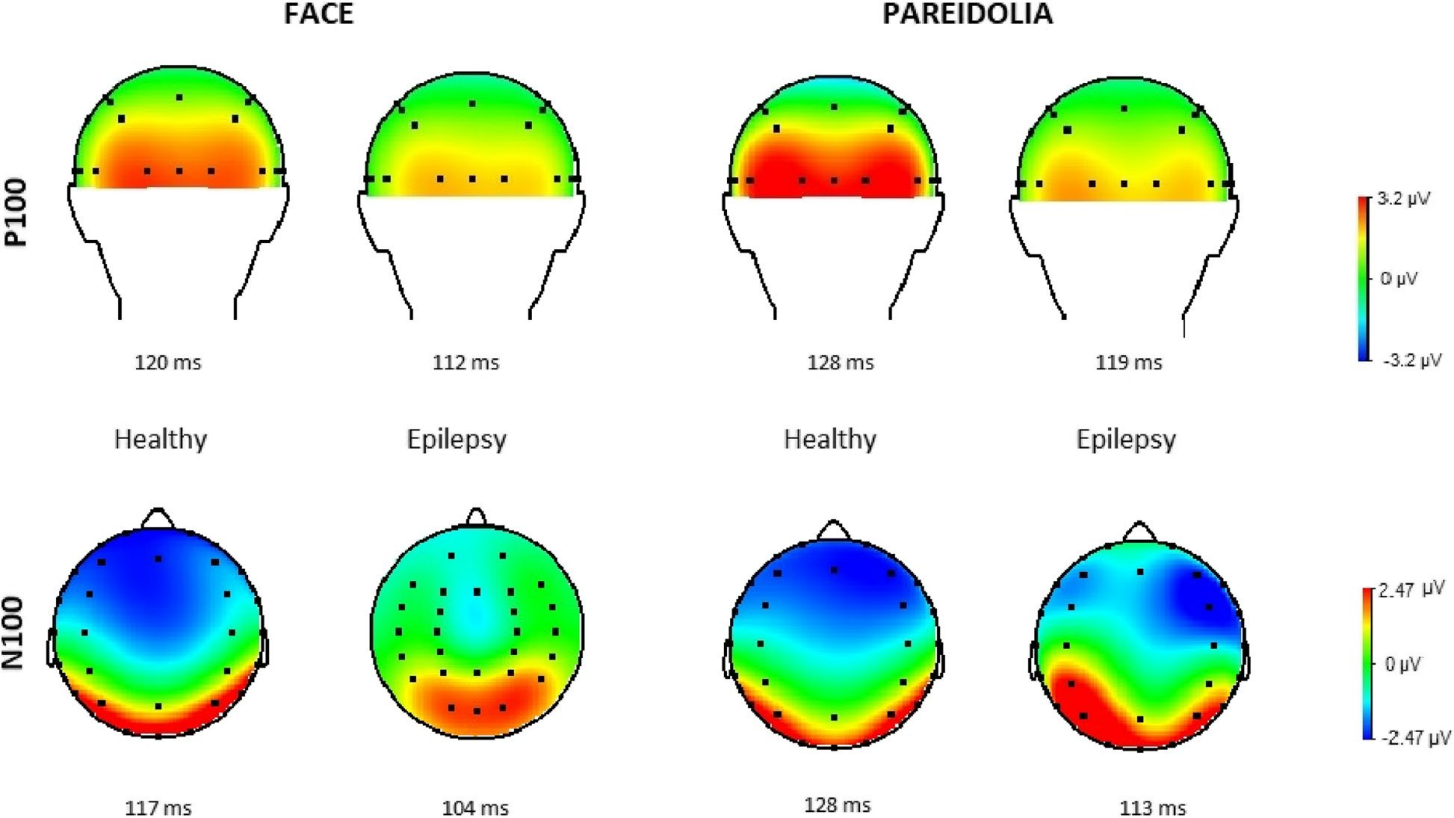
Current source density (CSD) maps around the P100 and N100 peak latencies during real-face and face-like perceptions. In the CSD maps of N100, the TLE patients had noticeable activity at the right anterior area in the pareidolia condition, but there was no difference in face condition between controls and TLE patients. In the CSD maps of P100, the TLE patients showed more diffuse source activity during the pareidolia condition. The TLE patients also showed stronger activation at the right posterior area in the pareidolia condition compared to the face condition for the N100 wave

EEGs were recorded in an isolated place using a 32-electrode cap (Brain Products GmbH, Gilching, ActiCHamp, Germany, impedance < 5 kΩ). The EEG signals were acquired using a computerized Brain Products system with a sampling rate of 1 kHz and a band-pass filter ranging from 0.16 Hz to 100 Hz. To monitor blinking and eye movements, bipolar electrooculographic (EOG) data were also recorded. The continuous EEG recordings were segmented into epochs, starting from 400 ms before to 1000 ms after the onset of the stimuli. A semi-automatic artifact rejection procedure was conducted to eliminate general artifacts present in all channels, as well as blinking artifacts in the EOG channel. Peak amplitudes within a time window of ± 30 ms were evaluated, which were centered at the maximum point of the grand average means. An automated peak detection function was used to identify the maximum and minimum peak amplitudes among N100 (fronto-central electrode area), P100 (occipitotemporal electrode area), N170 (occipitotemporal electrode area), and VPP (fronto-central electrode area) components. The analysis of each ERP component was performed at specific electrode sites as depicted in Fig. 2. The electrode sites were made according to the international 10–20 system. Furthermore, the peak amplitude and the latency of the ERP components were exported for further analysis. Current source density (CSD) analyses were conducted for brain mapping of face and pareidolia perception using the spherical spline procedure (Fig. 3).

### Statistical analysis

Data are presented as means ± standard error (SE) and analysed with the SPSS statistical software package (Version 12.0; SPSS Inc., Chicago, IL). Normality of data was assessed using the Shapiro–Wilk test. Levene's test was conducted to evaluate the homogeneity of variance. Independent samples* t*-test was used to compare latency and amplitude between epilepsy patients and healthy controls. Bonferroni correction was implemented on individual tests at each ERP component to reduce the probability of Type 1 error. To ensure consistency and conservatism, we applied the highest count of derived factors as the basis for the Bonferroni correction in all *t*-tests, resulting in a corrected significance level of α = 0.00625 (0.05/8). A *P*-value of less than 0.006 was considered statistically significant.

## Results

**Fig. 4:**
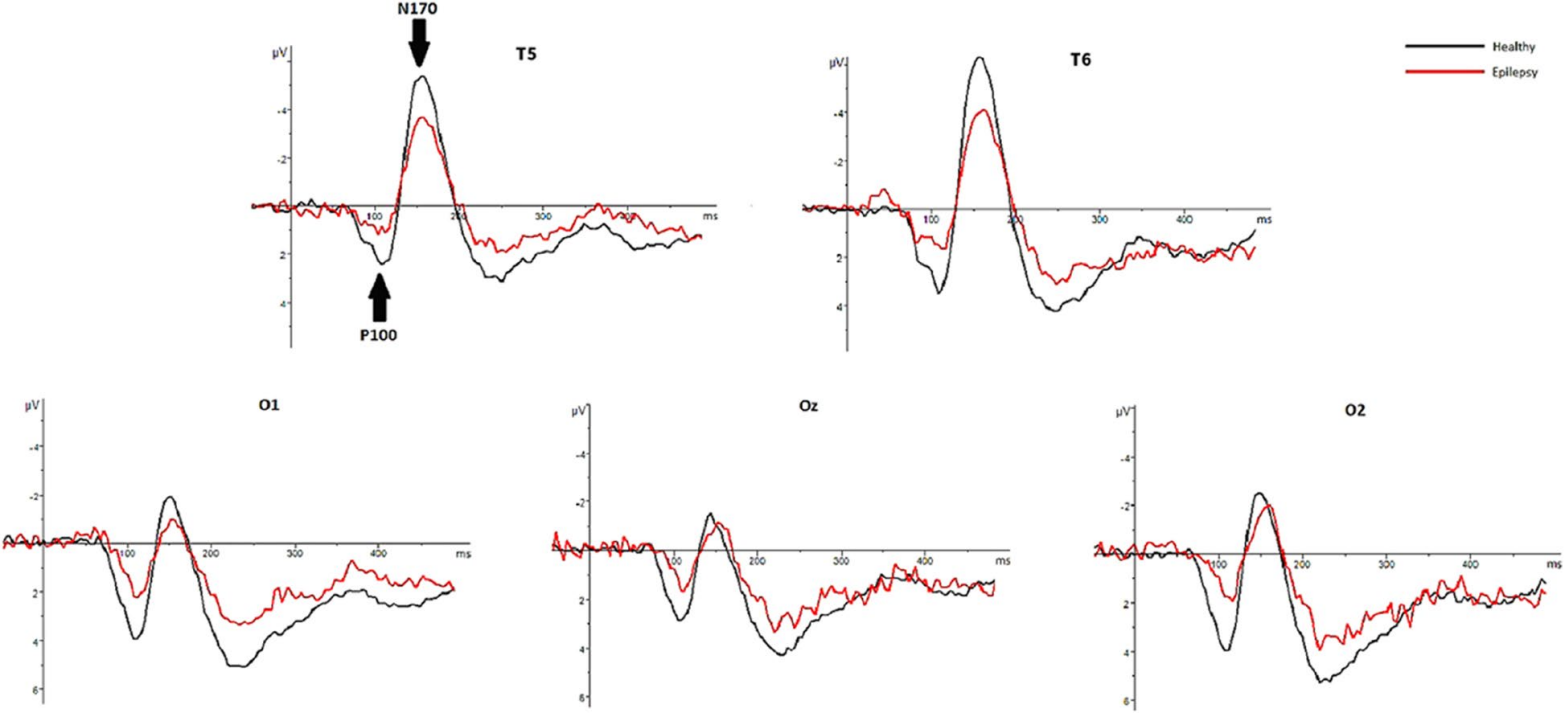
Graphs showing the grand average ERPs recorded from 28 patients with epilepsy (in red) and 30 healthy individuals (in black) in response to real-face images. Arrows indicate the P100 and N170 ERP components. The healthy individuals exhibited higher amplitudes than patients with epilepsy at around 100 ms and around 170 ms

**Fig. 5:**
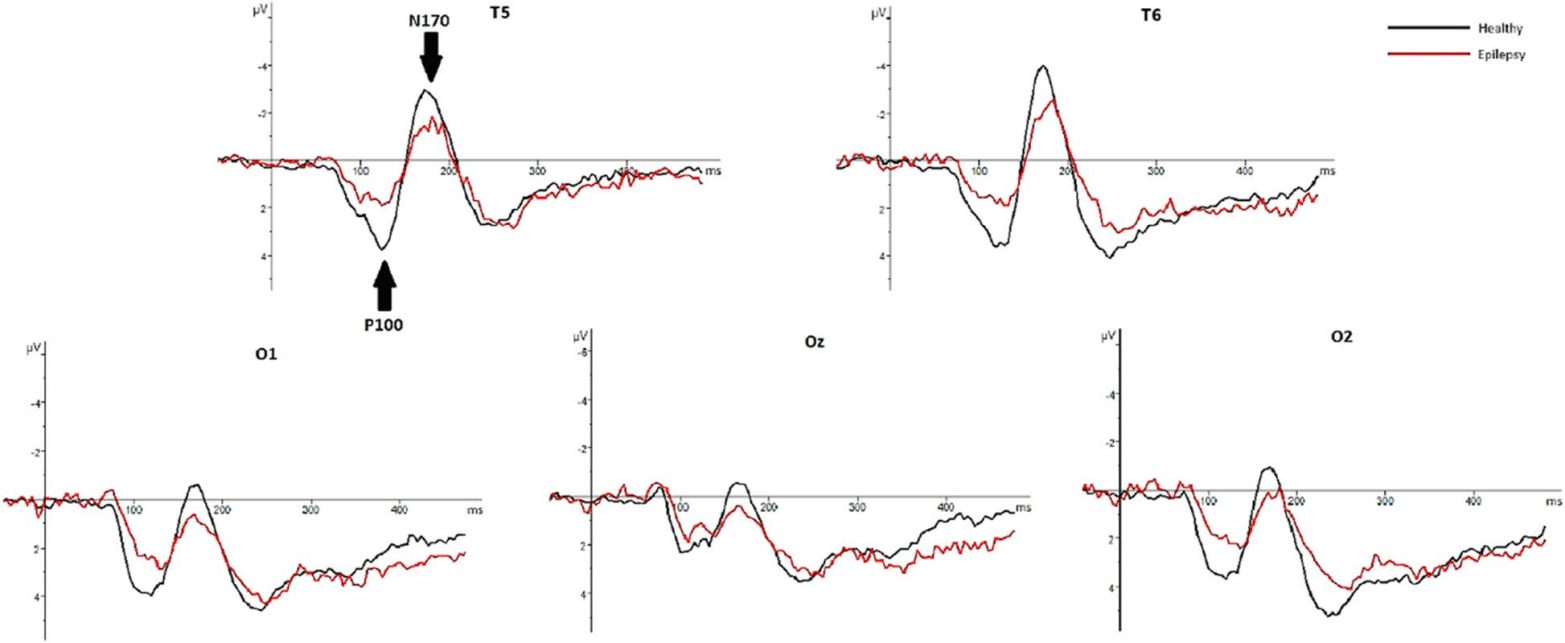
Graphs showing the grand average ERP recorded from epilepsy patients (in red) and healthy individuals (in black) in response to pareidolias face-like images. Arrows indicate the P100 and N170 ERP components. The healthy individuals exhibited higher amplitudes than patients with epilepsy at around 100 ms and around 170 ms

**Fig. 6:**
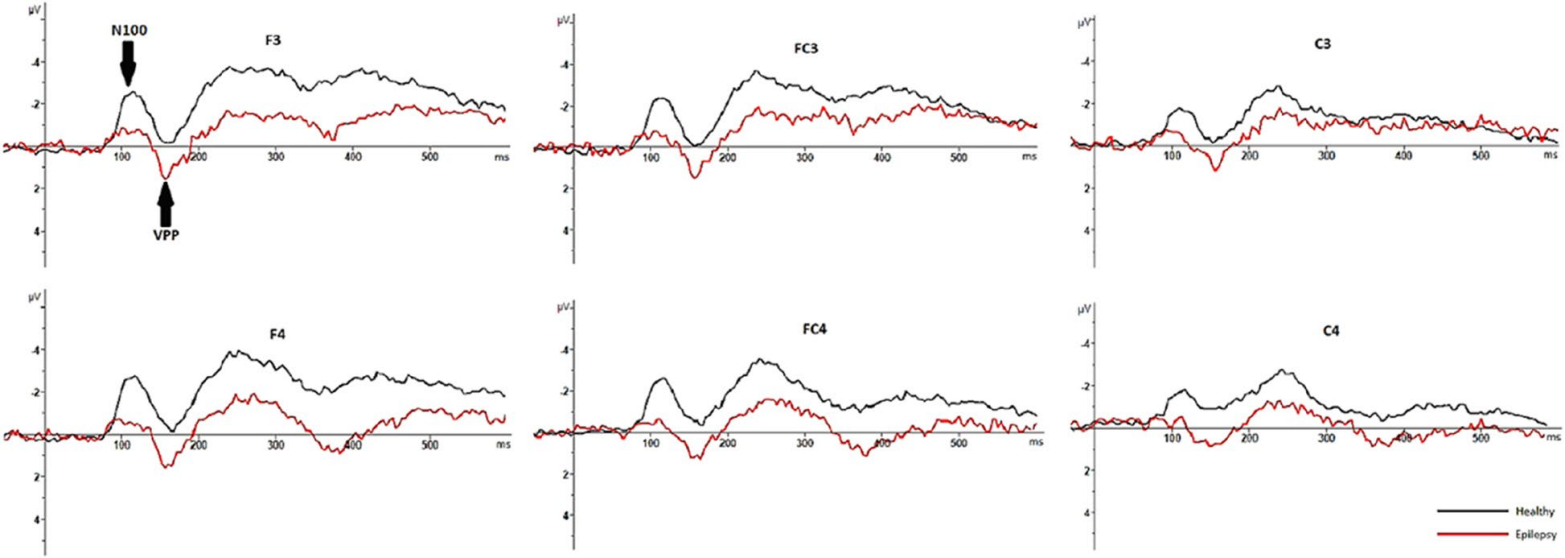
Graphs showing the grand average ERP recorded from 28 patients with epilepsy (in red) and 30 healthy (in black) in response to real-face images. Arrows indicate N100 and VPP components. The healthy individuals exhibited higher amplitude than patients with epilepsy at around 100 ms, while the patients with epilepsy exhibited higher amplitude than the healthy individuals at VPP

**Fig. 7:**
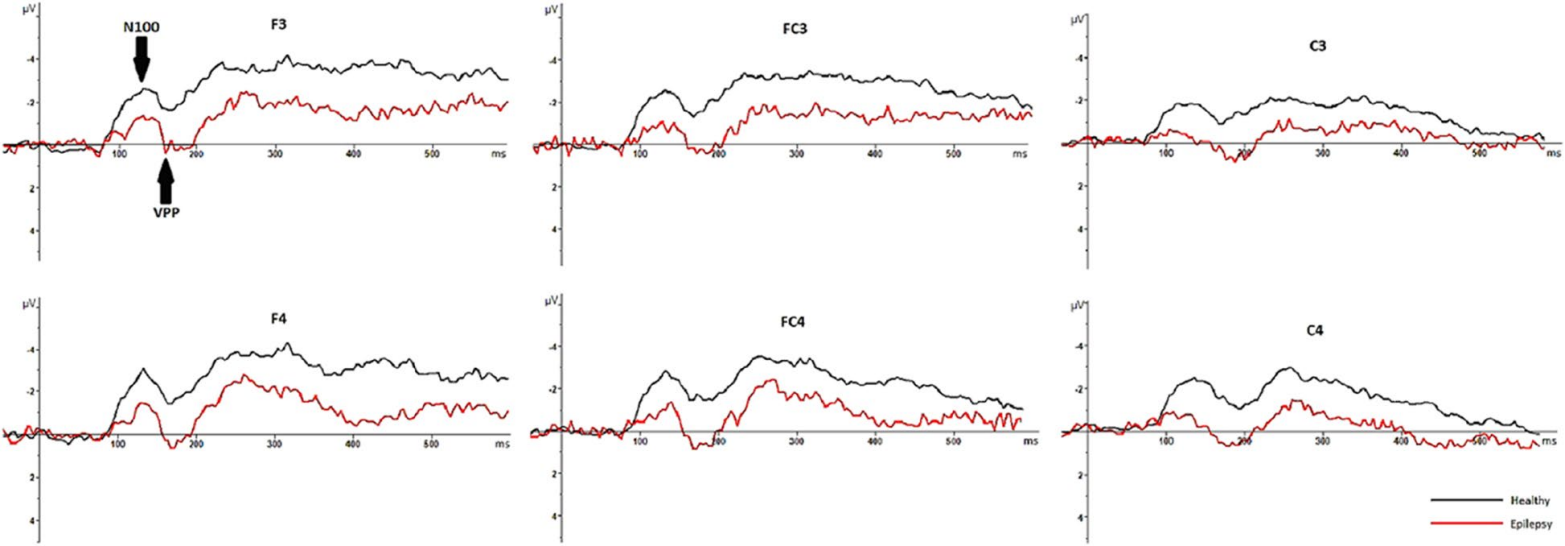
Graphs showing the grand average ERP recorded from epilepsy patients (in red) and healthy individuals (in black) in response to pareidolias face-like images. Arrows indicate the N100 and VPP components. The patients with epilepsy exhibited higher amplitudes than the healthy individuals at around 100 ms and at VPP

In healthy individuals, the mean latency to press the button in response to face and pareidolia images were 481 ± 43 ms and 579 ± 61 ms, respectively. In patients with epilepsy, the mean latency to respond to face and face-like images were 492 ± 16 ms and 591 ± 13 ms. The demographic and clinical characteristics of patients with epilepsy and healthy individuals is presented in Table 1. The mean and standard deviations (SD) of latencies and amplitudes of each ERP component are presented in Table 2. Figure 3 displays CSD maps in response to the face and face pareidolia stimulation in patients with TLE and healthy individuals within the time window of 90–150 ms. Grand-average waveforms representing the recorded brain activity in response to face and face pareidolia stimulation are depicted in Figs. 4, 5, 6 and 7. Notably, the P100, N100 and N170 components exhibited distinct early latency differential effects at posterior and anterior electrode sites. In this study, we focused on brain activity recorded within a time window of 90–190 ms.

**Table 2:** Means and standard deviations (SD) of latencies and amplitudes of ERP components in each group

			**TLE**		**Healthy**	
**ERP Component**			**Mean**	**SD**	**Mean**	**SD**
**N100**	Latency (ms)	Face Pareidolia	104.4593 113.4667	13.8908 18.7546	117.1010 128.6476	11.7359 12.1792
	Amplitude (μV)	Face Pareidolia	-1.5833 -2.1761	0.7216 1.1918	-2.3883 -2.6483	1.0793 1.3591
**N170**	Latency (ms)	Face Pareidolia	169.5704 174.6207	8.4590 8.5654	167.9226 172.9103	5.4703 7.3000
	Amplitude (μV)	Face Pareidolia	-3.2259 -1.9008	3.8470 2.8850	1.1478 3.4749	3.0580 3.2004
**P100**	Latency (ms)	Face Pareidolia	113.3913 120.2240	13.8526 16.0640	119.2471 127.2727	10.0961 11.9438
	Amplitude (μV)	Face Pareidolia	2.8959 4.2730	1.8353 2.1870	5.1017 5.6891	2.3723 2.6133
**VPP**	Latency (ms)	Face Pareidolia	171.9259 175.6552	8.9825 7.3546	169,.9048 173.4048	6.2426 6.8164
	Amplitude (μV)	Face Pareidolia	2.1268 1.4445	2.2937 1.8727	-0.1215 -1.0599	1.5220 1.3227

*TLE* Temporal lobe epilepsy. N100, VPP: F3, FC3, C3, F4, FC4, C4. N170, P100: T5, T6, O1, Oz, O2

### P100 (90–140 ms)

Analysis of P100 revealed a significant effect of stimuli category on the P100 latency in healthy participants. Specifically, face pareidolia perception elicited longer P100 latencies compared to face perception (*t*_54_ = -2.97, *P* = 0.001, d = 0.73). In epilepsy patients, face pareidolia perception induced higher amplitude and longer latency of P100 compared to face perception (*t*_58_ = -2.47, *P* = 0.001, d = 0.73; *t*_58_ = -2.37, *P* = 0.002, d = 0.52, respectively). Furthermore, the amplitudes of P100 for face pareidolia perception and face perception were higher in healthy participants than in epilepsy patients (*t*_56_ = 3.98, *P* = 0.000, d = 1.04; *t*_56_ = 2.18, *P* = 0.000, d = 0.62) (Figs. 4 and 5).

### N100 (100–150 ms)

In the control group, the latency of N100 was notably longer for face pareidolia perception than face perception (*t*_54_ = − 4.039, *P* = 0.000, d = 0.34). In epilepsy patients, face pareidolia perception induced higher amplitude and longer latency of N100 compared to face perception (*t*_54_ = 2.2, *P* = 0.002, d = 0.77; *t*_54_ = -2.03, *P* = 0.001, d = 0.98, respectively). Moreover, face perception evoked larger and longer N100 responses in healthy controls compared to epilepsy patients (*t*_56_ = -2.52, *P* = 0.002, d = 0.34; *t*_56_ = 3.88, *P* = 0.000, d = 0.77, respectively). Similarly, the amplitudes of N100 responses to face pareidolia perception were also larger and longer in healthy controls compared to epilepsy patients (*t*_56_ = -2.16, *P* = 0.001; *t*_56_ = 3.90, *P* = 0.001, respectively) (Figs. 6 and 7).

### N170 (160–190 ms)

Results of *t*-test analysis revealed significant differences in the amplitude and latency of the N170 component in healthy individuals between face and face pareidolia perceptions. Specifically, face pareidolia perception elicited higher amplitude and longer latency of N170 compared to face perception (*t*_54_ = -2.88,* P* = 0.002, d = 0.38; *t*_54_ = -3.01, *P* = 0.001, d = 0.42, respectively). In patients with epilepsy, face pareidolia perception induced a significantly longer latency of N170 compared to face perception (*t*_58_ = -2.22, *P* = 0.001, d = 0.38). Furthermore, both face pareidolia perception and face perception evoked larger N170 responses in healthy individuals compared to patients with epilepsy (*t*_56_ = 4.82, *P* = 0.000, d = 1.41; *t*_56_ = 6.72, *P* = 0.000, d = 0.90, respectively). However, there was no remarkable difference in the latency of the N170 component between healthy individuals and patients with epilepsy (Figs. 4 and 5). The average N170 component observed in this research exhibited a negative amplitude.

### VPP (160–190 ms)

There were significant differences in the VPP amplitude between epilepsy patients and healthy participants for both face and face pareidolia perceptions. Specifically, in response to real-face images, the VPP amplitude was significantly higher in epilepsy patients compared to healthy participants (*t*_54_ = 4.3, *P* = 0.000, d = 0.26). Similarly, in response to face pareidolia stimuli, the VPP amplitude was significantly higher in epilepsy patients compared to healthy participants (*t*_56_ = 6.22, *P* = 0.000, d = 1.56). However, there was no significant differences in VPP latency at the anterior sides between patients with epilepsy and healthy individuals for both face and face pareidolia stimuli (Figs. 6 and 7).

### CSD map

The CSD maps (Fig. 3) illustrate the brain activity during face perception and face pareidolia perception between 90 and 150 ms for both patients with TLE and healthy controls. During face pareidolia perception, patients with TLE exhibited strong activity at the right anterior area for N100 activity, while the control group displayed strong bilateral activity in the anterior area. During face perception, both the control group and the patients with TLE showed weaker activity at the posterior areas at P100 peak latency compared to that during face pareidolia perception. Furthermore, the source maps at the N100 peak latency were similar between the face perception and face pareidolia perception periods in the control group.

## Discussion

Despite the increasing research on the neural mechanisms underlying face perception in epilepsy, there are still many unanswered questions in this field. Our study was the first to investigate face pareidolia processing in epilepsy patients. To achieve this, we examined the N100, P100, N170, and VPP components of ERP. The latency of behavioural response to pareidolia images was longer than that to real-face images in the TLE group. Each ERP component exhibited similar latency between face and face pareidolia perception. Our findings revealed early indices that reflect differences in neural processing of real faces and face pareidolia in epilepsy.

In this study, we also observed significant differences in the P100, N100, N170, and VPP components in epilepsy patients compared to healthy participants in response to face-like and real-face images. Compared with healthy participants, patients with epilepsy had reduced P100, N100, and N170 amplitudes for both conditions.

Previous studies have indicated that the P100 component reflects the processing of low-level physical properties, such as dimension, brightness, color, and contrast of images [10, 34–36]. Furthermore, P100 affects the configuration of face processing [37, 38]. In our study, we observed attenuated P100 amplitudes in epilepsy patients for both face-like and real-face images. Also, the P100 amplitude was higher for face-like images compared to real-face images. We hypothesize that the higher amplitudes of P100 in response to face pareidolia than real faces may indicate a feature that distinguishes faces from objects in innate visual processing. Consistent with our suggestion, Itier and Taylor [10] claimed that the latency of P100 response was longer for inverted faces than upright faces, and this difference emerged from birth and persisted throughout the development of face recognition in visual processing. In our study, all images were converted to grey scale to control for low-level physical properties. The P100 latency is typically shorter in response to objects compared to actual face stimuli but longer in response to face-like stimuli [10, 21]. Our findings are in line with the literature, suggesting that epilepsy patients encode face and face pareidolia stimuli differently from the earliest phase of visual processing. Nihei et al. [39] reported similar P100 amplitudes for face and face-like stimuli, and our findings suggest disruption of this process in epilepsy. Generalized cognitive deficits observed in TLE suggest the presence of deficits in various brain regions, including the frontal lobe [40]. The observed difference in P100 amplitude in TLE patients in our study may be attributed to dysfunction in the frontal-temporal connections arising from seizures. Our study provides novel insights into the neural processing of face pareidolia in epilepsy patients.

In line with the findings for the P100 component, our study also revealed shorter latency and lower amplitude of the N100 component in epilepsy patients compared to the control group. This finding can be interpreted in two ways. First, we propose that the N100 component may be an inhibitory rebound from the preceding P100 component. The enhancement in the N100 amplitude following the face pareidolia stimulus may reflect an inhibitory rebound, suggesting that a stronger inhibitory rebound follows stronger activation when making decisions about the faces. It could be defined as a rebound effect where a negative component overlapped with the positive amplitude of the late positive component [41]. It could also be explained as the time when waveforms start to separate in a late time frame [42]. We claim that the inhibitory rebound plays a crucial role in modulating neural activity during face pareidolia tasks, as reflected in ERP components. As a result, this may support bottom-up processing of face pareidolia perception. Instead of considering them as separate indices, the pattern of the relationship between P100 and N100 may indicate a global deficit in visual processing. This suggests that the epilepsy patients may experience impairments in the early stages of visual processing, affecting both the P100 and the N100 components. Second, because N100 demonstrates sensitivity to emotional stimuli, the differences in N100 amplitude and latency in the epilepsy group compared to controls may reflect an alteration of affective processing in the patients [43]. However, since our study used neutral face stimuli devoid of emotional expressions, the difference found at N100 between the groups may be due to the negativity bias in epilepsy patients [44]. As observed in our experiment, while the controls perceived the stimuli as neutral faces, epilepsy patients may attribute negative emotions to the same stimuli, thereby resulting in the differences in the N100 component. These findings are intriguing and warrant further investigation. Since face pareidolia is not a real face, we analysed the N100 component for face pareidolia, which reflects object detection and recognition and potentially indicates the allocation of attention towards target stimuli such as a face or an object (cup of tea, clouds, etc.) during early processing of the ambiguous images. An important finding in this study is the larger N100 amplitude during face pareidolia perception than face perception. Future studies are needed to explore the underlying mechanisms and implications of the differences in the N100 component between epilepsy patients and healthy individuals. Additionally, it would be valuable to investigate the potential role of affective processing and negativity bias in epilepsy patients, as well as the impact of these factors on the perception of neutral stimuli.

In this study, we found larger occipital positivity for face pareidolia compared to real faces, reflected by larger amplitude P100 in the TLE group. We also found delayed latency of P100 for face pareidolia compared to faces, and delayed latency delay of N170 for face pareidolia. Our EEG analysis revealed a difference between the face and face pareidolia stimuli during the time period encompassing the P100 and N170 components. Supporting the hypothesis of Itier and Taylor [10], the return of the P100 wave component to the baseline during face pareidolia perception in the TLE group, suggests that the P100 face pareidolia is different from the N170 component. We hypothesize that early ERP component responses around 100 ms reflect the holistic perception of faces. This early response contributes to the face configuration recognition observed in the N170 response. The difference of the responses of the ERP components around 170 ms from the responses around 100 ms suggests that there is a problem in the configuration recognition of faces, not in the holistic recognition of the face in epilepsy patients [15].

The N170 potential is known to be particularly prominent during face detection and appears to operate at a configurable level. It is characterized by larger amplitudes and longer responses to face-like objects compared to actual faces, with the response amplitude being modulated by face resemblance [45]. A previous study found decreased N170 amplitudes in TLE patients compared to controls in response to inverted face images [30]. Consistent with these previous studies, our study found lower N170 amplitudes in epilepsy patients in response to both face and face pareidolia stimuli. The healthy controls showed larger amplitude and longer latency of N170 in response to face pareidolia stimuli compared to face stimuli. This finding suggests that the face pareidolia stimuli may lack the typical configuration of faces that is utilized in face processing. Based on these results, we propose that the processing of face pareidolia stimuli may differ from that of real faces. Face processing is mediated by specialized cognitive and neural mechanisms. If the face processing system is undergoing domain-specific development, there would be a greater increase in performance in face perception compared to other types of perceptions. Studies in this subject have yielded controversial results. Two studies have shown that brain regions that preferentially respond to facial stimuli experience a greater improvement than other functionally defined regions [46, 47]. Our hypothesis supports that face processing mechanisms are domain-specific for face stimuli and not process-specific for extracting configuration information [48].

We observed opposite patterns of amplitude change for N170 and VPP in epilepsy patients compared to healthy participants. Although the N170 and the VPP likely show identical neural mechanisms [21, 49], our findings indicate that the VPP amplitude was greater in the epilepsy group than in the healthy participants, suggesting a processing difference between these two components. Specifically, the VPP amplitude was greater in the epilepsy group compared to healthy participants, and the opposite for the N170 component. We propose that this discrepancy in VPP amplitude may be attributed to disruptions in the neural mechanisms involved in the structural encoding of faces in epilepsy. The VPP component, which was recorded from the frontal lobe, may involve executive function processes related to visual processing. This processing may additionally be impaired in epilepsy patients, resulting in hyperactivation during pareidolia perception and face processing. In addition, the VPP amplitude for the pareidolia stimuli was enhanced due to differential activation of the frontal lobe for face pareidolia stimuli compared to real faces. In support of this, Akdeniz et al. [50] investigated the effects of pareidolia and face stimuli on brain activation by functional magnetic resonance imaging (fMRI). They found that face pareidolia perception involves a significant role of the right prefrontal cortex as well as the inferior temporal region.

Leadner et al. [51] indicates that subcortical structures are involved in perception of face-like objects and real faces. Another study reported that these subcortical structures receive visual input possibly through early stages of visual system processing [52]. By integrating all this information, it becomes evident that these subcortical structures provide a developmental basis for facial pathways that will form the cortical network in adulthood, and impairment in these pathways may cause some developmental disorders [53]. It has been found that face-like pareidolia images are more difficult to detect than real faces in children with autism [54]. This study found for the first time that the delay in early visual processing in adult TLE patients is an indicator of impairment in the face and face-like pathways. The question of when and how this disorder occurs need to be investigated in future studies.

A limitation of the study is the lack of detailed examination of the cognitive and emotional states of participants, which can be further explored in follow-up studies.

## Conclusions

In conclusion, an early deficit in visual processing may account for differences in pareidolia and face processing in epilepsy. Furthermore, the changes in multiple early components of the ERP may reflect a global impairment in early visual processing in epilepsy. Despite some similarities in the processing of pareidolia and face stimuli, our results indicate distinct neural mechanisms underlying the processing of these stimuli in TLE. In future studies, advanced imaging techniques should be used to investigate the neural mechanisms involved in face pareidolia. Our study provided valuable information on the functioning of the visual system in epilepsy and may have implications for the diagnosis and treatment of visual processing deficits in this population.

## Data Availability

Data will be made available on request.

## Abbreviations

CSD: Current source density
EEG: Electroencephalography
ERP: Event-related potentials
EOG: Electrooculographic
FFA: Fusiform face area
fMRI: Functional magnetic resonance imaging
HS: Hippocampal sclerosis
TLE: Temporal lobe epilepsy
VPP: Vertex positive potential
